# Supplementary material for: A broad-host-range Rhizobium rhizogenes strain enables transient expression across diverse crop species
Source: PLoS Biol. 2026 Oct 15;24(10):e3004010. doi: 10.1371/journal.pbio.3004010 (PMC13671559; doi:10.1371/journal.pbio.3004010)

Fig 2H

GFP RT-PCR in faba bean

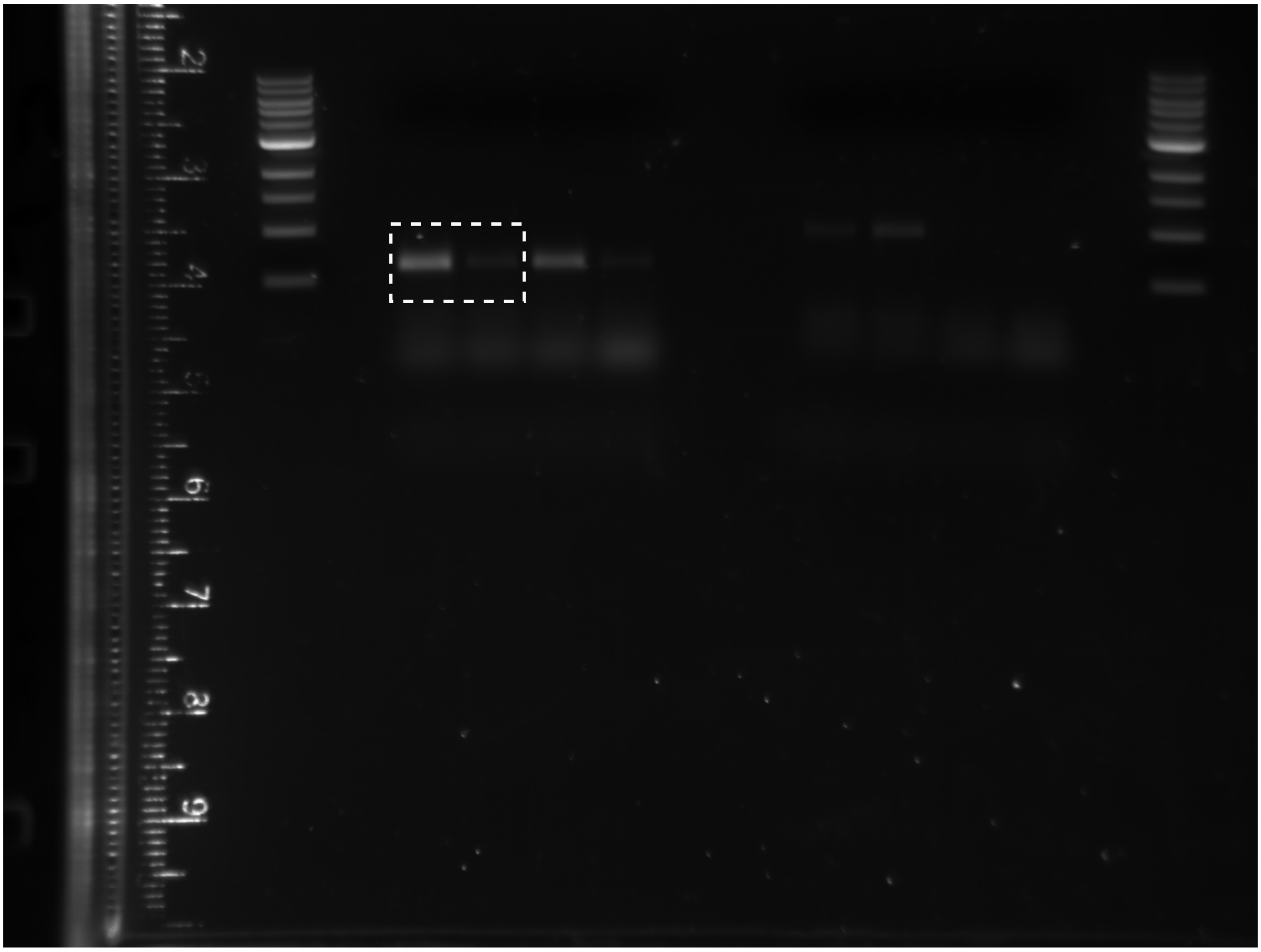

GAPDH RT-PCR in faba bean

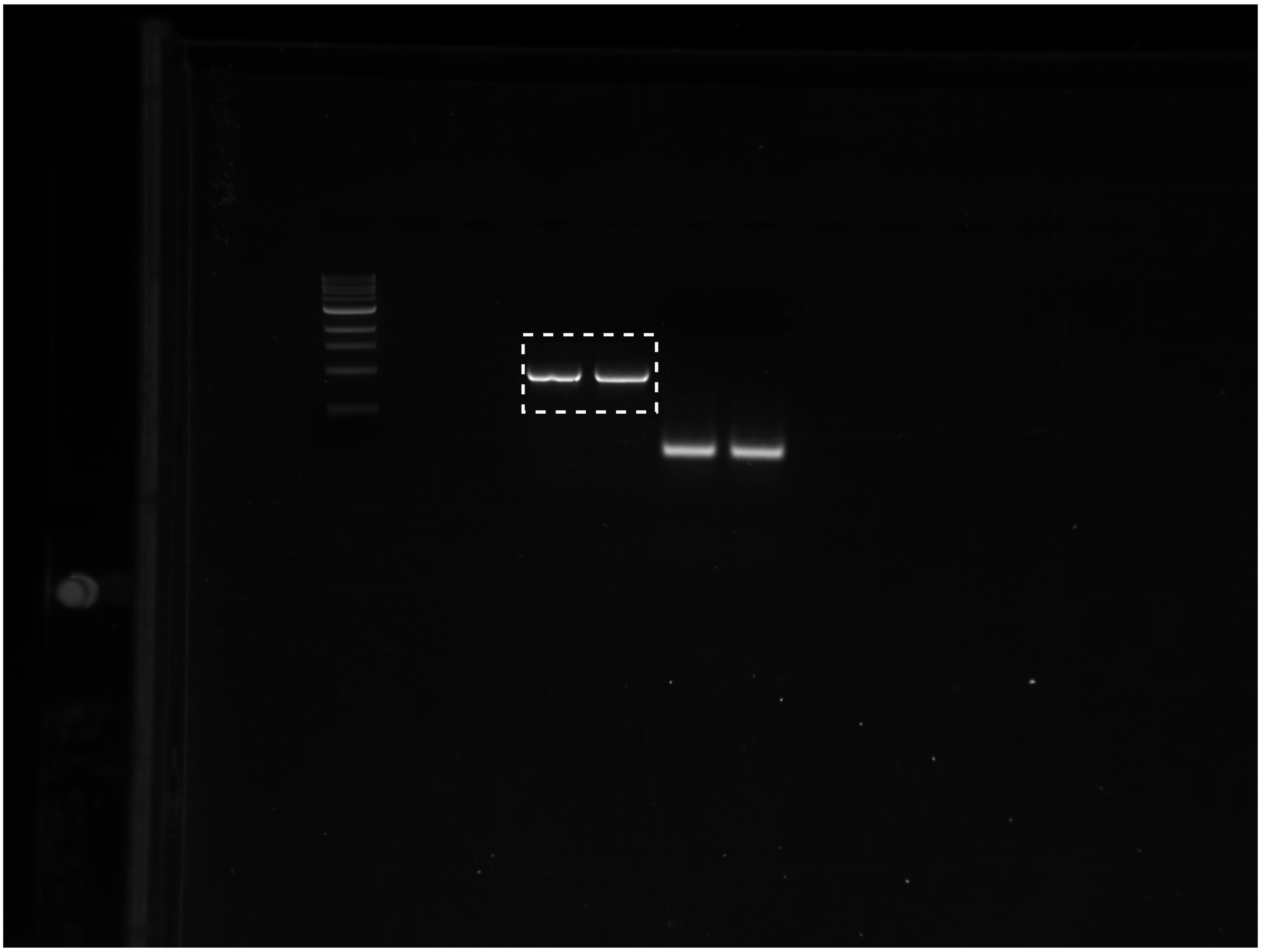

Fig S5G

RT-PCR GAPDH in lettuce

RT-PCR GFP in lettuce

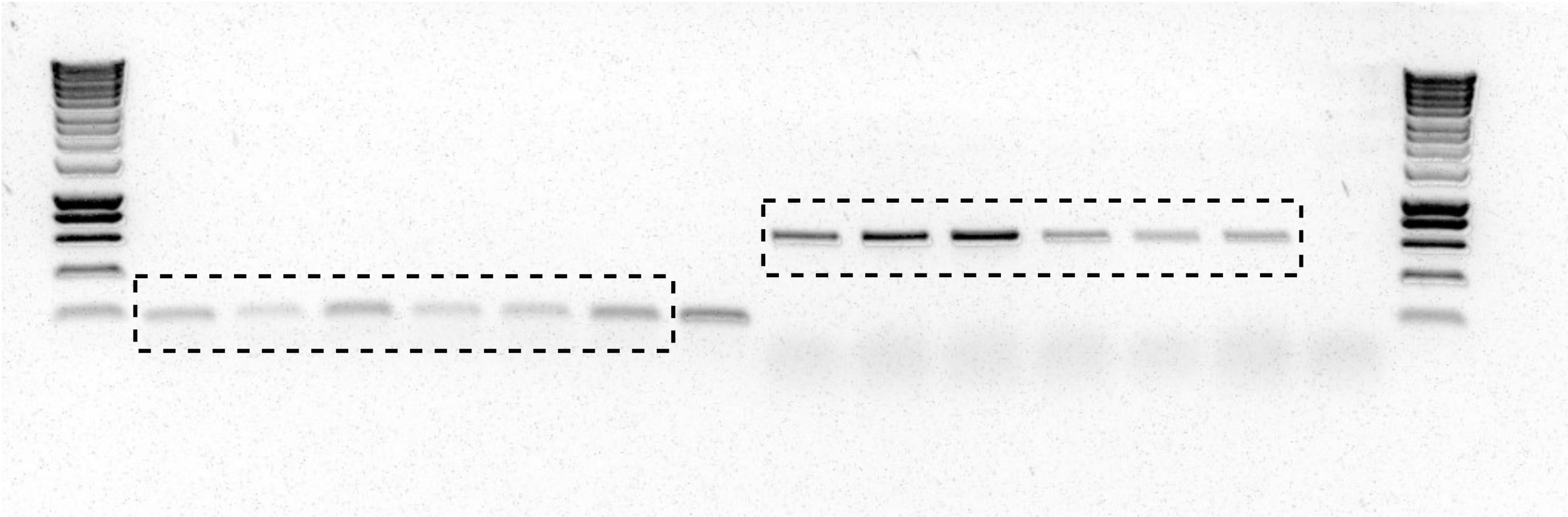

Supplement: Supplementary file 1 [file pbio.3004010.s013.pdf]
