# Supplementary material for: A broad-host-range Rhizobium rhizogenes strain enables transient expression across diverse crop species
Source: PLoS Biol. 2026 Oct 15;24(10):e3004010. doi: 10.1371/journal.pbio.3004010 (PMC13671559; doi:10.1371/journal.pbio.3004010)
Supplement: Supplementary file 2 [file pbio.3004010.s008.docx]

| **Species** | **Family** | **Cultivar(s)** | **Source(s)** |
| --- | --- | --- | --- |
| *Petunia hybrida* | Solanaceae | W115 | Academia Sinica transformation core |
| *Nicotiana tabacum* | Solanaceae | Samsun; Xanthi | Kindly donated by Ya-Chun Chang (National Taiwan University) |
| *Solanum tuberosum* | Solanaceae | Kennebec | Local supermarket |
| *S. nigrum* | Solanaceae | NA | Wild accession |
| *Lycium barbarum* | Solanaceae | NA | Local commercial supplier |
| *Physalis peruviana* | Solanaceae | NA | Local supermarket |
| *Ipomoea alba* | Convolvulaceae | NA | Local commercial supplier |
| *I. hederacea* | Convolvulaceae | NA | Wild accession |
| *I. aquatica* | Convolvulaceae | NA | Local commercial supplier |
| *I. batatas* | Convolvulaceae | Taoyuan No. 2 | Local commercial supplier |
| *Mimulus lewisii* | Phrymaceae | LF10 | Kindly donated by Chun-Neng Wang  (National Taiwan University) |
| *Salvia splendens* | Lamiaceae | NA | Commercial supplier (Known-You Seed) |
| *Antirrhinum majus* | Plantaginaceae | NA | Commercial supplier (Known-You Seed) |
| *Sesamum indicum* | Pedaliaceae | NA | Local commercial supplier |
| *Coffea canephora* | Rubiaceae | NA | Local commercial supplier |
| *Lactuca sativa* | Asteraceae | NA; Amica | Commercial supplier (Known-You Seed); Commercial supplier (Enza Zaden) |
| *Chenopodium formosanum* | Amaranthaceae | NA | Kindly donated by Na-Sheng Lin (IPMB Academia Sinica) |
| *Brassica rapa* | Brassicaceae | Standard 1-33 | Commercial supplier (Rapid-Cycling Brassica Collection) |
| *Medicago sativa* | Fabaceae | NA | Local commercial supplier |
| *Trigonella foenum-graecum* | Fabaceae | NA | Local commercial supplier |
| *Glycine max* | Fabaceae | Kaohsiung-11 | Local commercial supplier |
| *Pisum sativum* | Fabaceae | Jin Jin | Local commercial supplier |
| *Vicia faba* | Fabaceae | NA; de Monica | Local commercial supplier; commercial supplier (Thompson-Morgan) |
| *Phaseolus vulgaris* | Fabaceae | NA | Commercial supplier (Known-You Seed) |
| *Vigna unguiculata* | Fabaceae | NA | Local commercial supplier |
| *V. radiata* | Fabaceae | Crystal | Kindly donated by Heng-Ming Ting  (National Taiwan University) |
