# Supplementary material for: A broad-host-range Rhizobium rhizogenes strain enables transient expression across diverse crop species
Source: PLoS Biol. 2026 Oct 15;24(10):e3004010. doi: 10.1371/journal.pbio.3004010 (PMC13671559; doi:10.1371/journal.pbio.3004010)
Supplement: Supplementary file 3 [file pbio.3004010.s009.docx]

| **Elicitor name** | **Pathogen** | **Genbank Gene Accession** | **Reference** |
| --- | --- | --- | --- |
| *Bc*CDI1 | *Botrytis cinerea* | XP_001560184.1 | [1] |
| *Bc*pg1 | *B. cinerea* | XP_001550077.1 | [2] |
| *Bc*xyn11A | *B. cinerea* | XP_024547454.1 | [3] |
| *Cc*EG1 | *Cytospora chrysosperma* | ROV90154.1 | [4] |
| *Cf*ECP2 | *Cladosporium fulvum* | CAA78401.1 | [5] |
| *Cs*02526 | *Ciboria shiraiana* | [WLS32030.](https://www.ncbi.nlm.nih.gov/nuccore/WLS32030.1)1 | [6] |
| *Ds*70057 | *Dothistroma septosporum* | EME45906.1 | [7] |
| *Ds*71487 | *D. septosporum* | EME45809.1 | [7] |
| *Ds*74283 | *D. septosporum* | EME40684.1 | [7] |
| *Fg*62 | *Fusarium graminearum* | XP_011320562.1 | [8] |
| *Gr*EXPB2 | *Globodera rostochiensis* | AJ311902.1 | [9] |
| *Gr*PEL2 | *G.rostochiensis* | AY094613.1 | [10] |
| *Aa*HRIP1 | *Alternaria alternata* | HQ713431.1 | [11] |
| *Zt*NLP | *Zymoseptoria tritici* | SMQ56213.1 | [12] |
| *Xf*PD_0956 | *Xylella fastidiosa* | AAO28818.1 | [13] |
| PITG_23123 (SCR50) | *Phytophthora infestans* | XP_002997761.1 | [14] |
| *Rc*CDI1 | *Rhynchosporium commune* | ARB51385.1 | [1] |
| *Ss*NE1 (SS1G_07027) | *Sclerotinia sclerotiorum* | XP_001591581 | [15] |
| *Ss*NE2 (SS1G_00849) | *S. sclerotiorum* | XP_001598760 | [15] |
| *Ss*Xyl2 (SS1G_03618) | *S. sclerotiorum* | XP_001595529.1 | [16] |
| *Vd*EG1 (VDAG_04017) | *Verticillium dahliae* | XM_009654101.1 | [17] |
| *Zt*10 (MYCGRDRAFT_111505) | *Z. tritici* | EGP82940.1 | [18] |
| *Ps*XEG1 | *P. sojae* | G4ZHR2.1 | [19] |

**References**

1. Franco-Orozco B, Berepiki A, Ruiz O, Gamble L, Griffe LL, Wang S, et al. A new proteinaceous pathogen-associated molecular pattern (PAMP) identified in Ascomycete fungi induces cell death in Solanaceae. New Phytol. 2017;214(4):1657-72.
2. Ji D, Liu W, Cui X, Liu K, Liu Y, Huang X, et al. A receptor-like kinase SlFERL mediates immune responses of tomato to Botrytis cinerea by recognizing BcPG1 and fine-tuning MAPK signaling. New Phytol. 2023;240(3):1189-201.
3. Frias M, Gonzalez M, Gonzalez C, Brito N. A 25-Residue Peptide From Botrytis cinerea Xylanase BcXyn11A Elicits Plant Defenses. Front Plant Sci. 2019;10:474.
4. Xu ZY, Luo Z, Xiong DG, Gao M, Tian CM. A glycoside hydrolase 12 protein from triggers plant immunity but is not essential to virulence. Phytopathol Res. 2023;5(1).
5. Van den Ackerveken GF, Van Kan JA, Joosten MH, Muisers JM, Verbakel HM, De Wit PJ. Characterization of two putative pathogenicity genes of the fungal tomato pathogen Cladosporium fulvum. Mol Plant Microbe Interact. 1993;6(2):210-5.
6. Zhang S, Li R, Fan W, Chen X, Liu S, Zhu P, et al. Effector Cs02526 from Ciboria shiraiana induces cell death and modulates plant immunity. Plant Physiol. 2024;196(1):579-91.
7. Hunziker L, Tarallo M, Gough K, Guo M, Hargreaves C, Loo TS, et al. Apoplastic effector candidates of a foliar forest pathogen trigger cell death in host and non-host plants. Sci Rep. 2021;11(1):19958.
8. Wang SC, Yang S, Dai KX, Zheng WY, Zhang XY, Yang B, et al. The effector Fg62 contributes to virulence and induces plant cell death. Phytopathol Res. 2023;5(1).
9. Ali S, Magne M, Chen SY, Côté O, Stare BG, Obradovic N, et al. Analysis of Putative Apoplastic Effectors from the Nematode, and Identification of an Expansin-Like Protein That Can Induce and Suppress Host Defenses. Plos One. 2015;10(1).
10. Kudla U, Milac AL, Qin L, Overmars H, Roze E, Holterman M, et al. Structural and functional characterization of a novel, host penetration-related pectate lyase from the potato cyst nematode Globodera rostochiensis. Mol Plant Pathol. 2007;8(3):293-305.
11. Jones RW, Perez F. Differential plant response to toxins and elicitor proteins released by the potato and tomato pathogens Alternaria solani and Alternaria alternata. J Plant Pathol. 2023;105(1):21-8.
12. Motteram J, Kufner I, Deller S, Brunner F, Hammond-Kosack KE, Nurnberger T, et al. Molecular characterization and functional analysis of MgNLP, the sole NPP1 domain-containing protein, from the fungal wheat leaf pathogen Mycosphaerella graminicola. Mol Plant Microbe Interact. 2009;22(7):790-9.
13. Sertedakis M, Kotsaridis K, Tsakiri D, Mermigka G, Dominguez-Ferreras A, Ntoukakis V, et al. Expression of putative effectors of different Xylella fastidiosa strains triggers cell death-like responses in various Nicotiana model plants. Mol Plant Pathol. 2022;23(1):148-56.
14. Bos JIB, Armstrong M, Whisson SC, Torto TA, Ochwo M, Birch PRJ, et al. Intraspecific comparative genomics to identify avirulence genes from Phytophthora. New Phytol. 2003;159(1):63-72.
15. Seifbarghi S, Borhan MH, Wei Y, Ma L, Coutu C, Bekkaoui D, et al. Receptor-Like Kinases BAK1 and SOBIR1 Are Required for Necrotizing Activity of a Novel Group of Sclerotinia sclerotiorum Necrosis-Inducing Effectors. Front Plant Sci. 2020;11:1021.
16. Wang P, Wang Y, Hu Y, Chen Z, Han L, Zhu W, et al. Plant hypersensitive induced reaction protein facilitates cell death induced by secreted xylanase associated with the pathogenicity of Sclerotinia sclerotiorum. Plant J. 2024;118(1):90-105.
17. Gui YJ, Chen JY, Zhang DD, Li NY, Li TG, Zhang WQ, et al. Verticillium dahliae manipulates plant immunity by glycoside hydrolase 12 proteins in conjunction with carbohydrate-binding module 1. Environ Microbiol. 2017;19(5):1914-32.
18. Kettles GJ, Bayon C, Canning G, Rudd JJ, Kanyuka K. Apoplastic recognition of multiple candidate effectors from the wheat pathogen Zymoseptoria tritici in the nonhost plant Nicotiana benthamiana. New Phytol. 2017;213(1):338-50.
19. Ma Z, Song T, Zhu L, Ye W, Wang Y, Shao Y, et al. A Phytophthora sojae Glycoside Hydrolase 12 Protein Is a Major Virulence Factor during Soybean Infection and Is Recognized as a PAMP. Plant Cell. 2015;27(7):2057-72.
