# Supplementary material for: A broad-host-range Rhizobium rhizogenes strain enables transient expression across diverse crop species
Source: PLoS Biol. 2026 Oct 15;24(10):e3004010. doi: 10.1371/journal.pbio.3004010 (PMC13671559; doi:10.1371/journal.pbio.3004010)
Supplement: Supplementary file 4 [file pbio.3004010.s010.docx]

| **Oligos for cloning *Vf*CC-NLR into level 0 from faba bean cDNA** | |
| --- | --- |
| **Name** | **Sequence** |
| VfRx-CNL GG F2 | CACTCTGTGGTCTCAAATGGCAGAAACTACAGTGTC |
| VfRx-CNL GG R2 | ATTCGTGGTCTCACGAACCGACATTGAGTTGAGTAGGAATCG |
| **Oligos for D512V mutagenesis of *Vf*CC-NLR** | |
| **Name** | **Sequence** |
| VfRx-CNL D512V F | TCAAGTCCATGTGTTGTTGCGCGAAG |
| VfRx-CNL D512V R | CAACTTTTCACTTTCCCATCAAAAC |
| **Oligos for H480A mutagenesis of *Nb*NRC2** | |
| **Name** | **Sequence** |
| SB_NbNRC2_H480A_F1 | GACATGTTGCACGAGTTTTGCAGACAA |
| SB_NbNRC2_H480A_R1 | AGCAAGACGACATGTTTTGATTTGACC |
| **Oligos for RT-PCR of GFP** | |
| **Name** | **Sequence** |
| GFP RT-PCR F7 | CGACGTAAACGGCCACAAGTTC |
| GFP RT-PCR R2 | GCTCGTCCATGCCGAGAG |
| **Oligos for RT-PCR of *Vf*GAPDH** | |
| **Name** | **Sequence** |
| VfGAPDH RT-PCR F3 | CCAAGATCAAGATCGGAATCAAC |
| VfGAPDH RT-PCR R4 | GGTCAACCACACGGGTACTG |
| **Oligos for RT-PCR of *Ls*GAPDH** | |
| **Name** | **Sequence** |
| Ls_GAPDH_qPCR_F3 | CACGATCATCAGCAACGCCT |
| Ls_GAPDH_qPCR_R3 | GGACGATGTTCAGTGCTGCA |
