# Supplementary material for: A broad-host-range Rhizobium rhizogenes strain enables transient expression across diverse crop species
Source: PLoS Biol. 2026 Oct 15;24(10):e3004010. doi: 10.1371/journal.pbio.3004010 (PMC13671559; doi:10.1371/journal.pbio.3004010)
Supplement: Supplementary file 5 [file pbio.3004010.s011.docx]

| **Construct** | **Parts** | **Reference** |
| --- | --- | --- |
| p35S::RUBY::tOCS (pICSL86922OD) | / | [1] |
| p35S::RUBY::t35S (pJK001c) | pICH51288, pFK1S-0331, pICSL50028, pICH41414, pJK001c | This study |
| p35S::eGFP::t35S | pICH51288, eGFP pTwist, pICH41414, pJK001c | This study |
| p35S::mCherry::t35S | / | [2] |
| eGFP-pRNAi | / | [3] |
| GUS-pRNAi | / | [4] |
| pMas::*Vf*CC-NLR^WT^-GFP::tHSP | pICH8521, pFK1S-0176, pICSL50034, pICSL60008, pJK001c | This study |
| pMas::*Vf*CC-NLR^D512V^-GFP::tHSP | pICH8521, pFK1S-0177, pICSL50034, pICSL60008, pJK001c | This study |
| pMas::*Nb*NRC2A^WT^-GFP::tHSP | pICH8521, pJK-0-116, pICSL50034, pICSL60008, pJK001c | This study |
| p35S::*Nb*NRC2A^H480A^::t35S | pICH51288, SB-NbNRC2-H480A, pICSL50028, pICH41414, pJK001c | This study |
| pMas::*Sl*NRC3^WT^-GFP::tHSP | pICH8521, pJK-0-224, pICSL50034, pICSL60008, pJK001c | This study |
| pMas::*Sl*NRC3^D480V^-GFP::tHSP | pICH8521, pJK-0-225, pICSL50034, pICSL60008, pJK001c | This study |
| pMas::*Nb*NRC4^WT^-GFP::tHSP | pICH8521, pFK1S-0005, pICSL50034, pICSL60008, pJK001c | This study |
| pMas::*Nb*NRC4^D478V^-GFP::tHSP | pICH8521, pJK-0-435, pICSL50034, pICSL60008, pJK001c | This study |
| pMas::*Nb*NRC4^L9E/V10E/L14E^-GFP::tHSP | pICH8521, pJK-0-245, pICSL50034, pICSL60008, pJK001c | This study |
| pMas::*Nb*NRC4^L9E/V10E/L14E/D478V^-GFP::tHSP | pICH8521, pJK-0-436, pICSL50034, pICSL60008, pJK001c | This study |
| pMas::*Nb*NRC4^L9E/D478V^-GFP::tHSP | pICH8521, pFK1S-0383, pICSL50034, pICSL60008, pJK001c | This study |
| pRpi-vnt1::Rpi-vnt1::tRpi-vnt1 | / | [5] |
| p35S::RFP-Avr-vnt1::t35S | / | [6] |
| p35S::Rx-BFP::t35S | ccdB-BFP pK7WGF2 | This study |
| p35S::PVX CP-Myc::t35S | / | [7] |
| p35S::RFP-StREMORIN1.3::t35S | / | [8] |
| p35S::*Nt*PR1a-SP::*Bc*CDI1::tHSP | pJK001c, pICH51226, pJK-NT-0017, pNE-CDS-0001, pICSL50028, pICSL6008 | This study |
| p35S::*Nt*PR1a-SP::*Bc*pg1::tHSP | pJK001c, pICH51226, pJK-NT-0017, pNE-CDS-0003, pICSL50028, pICSL6008 | This study |
| p35S::*Nt*PR1a-SP::*Bc*xyn11A::tHSP | pJK001c, pICH51226, pJK-NT-0017, pNE-CDS-0006, pICSL50028, pICSL6008 | This study |
| p35S::*Nt*PR1a-SP::*Cc*EG1::tHSP | pJK001c, pICH51226, pJK-NT-0017, pNE-CDS-0007, pICSL50028, pICSL6008 | This study |
| p35S::*Nt*PR1a-SP::*Cf*ECP2::tHSP | pJK001c, pICH51226, pJK-NT-0017, pNE-CDS-0008, pICSL50028, pICSL6008 | This study |
| p35S::*Nt*PR1a-SP::*Cs*02526::tHSP | pJK001c, pICH51226, pJK-NT-0017, pNE-CDS-0009, pICSL50028, pICSL6008 | This study |
| p35S::*Nt*PR1a-SP::*Ds*70057::tHSP | pJK001c, pICH51226, pJK-NT-0017, pNE-CDS-0010, pICSL50028, pICSL6008 | This study |
| p35S::*Nt*PR1a-SP::*Ds*71487::tHSP | pJK001c, pICH51226, pJK-NT-0017, pNE-CDS-0011, pICSL50028, pICSL6008 | This study |
| p35S::*Nt*PR1a-SP::*Ds*74283::tHSP | pJK001c, pICH51226, pJK-NT-0017, pNE-CDS-0012, pICSL50028, pICSL6008 | This study |
| p35S::*Nt*PR1a-SP::*Fg*62::tHSP | pJK001c, pICH51226, pJK-NT-0017, pNE-CDS-0014, pICSL50028, pICSL6008 | This study |
| p35S::*Nt*PR1a-SP::*Gr*EXPB2::tHSP | pJK001c, pICH51226, pJK-NT-0017, pNE-CDS-0015, pICSL50028, pICSL6008 | This study |
| p35S::*Nt*PR1a-SP::*Gr*PEL2::tHSP | pJK001c, pICH51226, pJK-NT-0017, pNE-CDS-0017, pICSL50028, pICSL6008 | This study |
| p35S::*Nt*PR1a-SP::*Aa*HRIP1::tHSP | pJK001c, pICH51226, pJK-NT-0017, pNE-CDS-0018, pICSL50028, pICSL6008 | This study |
| p35S::*Nt*PR1a-SP::*Zt*NLP::tHSP | pJK001c, pICH51226, pJK-NT-0017, pNE-CDS-0019, pICSL50028, pICSL6008 | This study |
| p35S::*Nt*PR1a-SP::*Xf*PD_0956::tHSP | pJK001c, pICH51226, pJK-NT-0017, pNE-CDS-0021, pICSL50028, pICSL6008 | This study |
| p35S::*Nt*PR1a-SP::PITG_23123::tHSP | pJK001c, pICH51226, pJK-NT-0017, pNE-CDS-0024, pICSL50028, pICSL6008 | This study |
| p35S::*Nt*PR1a-SP::*Rc*CDI1::tHSP | pJK001c, pICH51226, pJK-NT-0017, pNE-CDS-0025, pICSL50028, pICSL6008 | This study |
| p35S::*Nt*PR1a-SP::*Ss*NE1::tHSP | pJK001c, pICH51226, pJK-NT-0017, pNE-CDS-0027, pICSL50028, pICSL6008 | This study |
| p35S::*Nt*PR1a-SP::*Ss*NE2::tHSP | pJK001c, pICH51226, pJK-NT-0017, pNE-CDS-0028, pICSL50028, pICSL6008 | This study |
| p35S::*Nt*PR1a-SP::*Ss*Xyl2::tHSP | pJK001c, pICH51226, pJK-NT-0017, pNE-CDS-0031, pICSL50028, pICSL6008 | This study |
| p35S::*Nt*PR1a-SP::*Vd*EG1::tHSP | pJK001c, pICH51226, pJK-NT-0017, pNE-CDS-0032, pICSL50028, pICSL6008 | This study |
| p35S::*Nt*PR1a-SP::*Zt*10::tHSP | pJK001c, pICH51226, pJK-NT-0017, pNE-CDS-0037, pICSL50028, pICSL6008 | This study |
| pMas::*Nt*PR1a-SP::*Ps*XEG1::tHSP pJK001c | pICH8521, *Ps*XEG1 pTwist, pICSL50028, pICSL60008, pJK001c | This study |

**References**

1. Lopez-Agudelo JC, Goh FJ, Tchabashvili S, Huang YS, Huang CY, Lee KT, et al. Rhizobium rhizogenes A4-derived strains mediate hyper-efficient transient gene expression in Nicotiana benthamiana and other solanaceous plants. Plant Biotechnol J. 2026;24(1):131-44.
2. Kourelis J, Contreras MP, Harant A, Pai H, Ludke D, Adachi H, et al. The helper NLR immune protein NRC3 mediates the hypersensitive cell death caused by the cell-surface receptor Cf-4. PLoS Genet. 2022;18(9):e1010414.
3. Yan P, Shen W, Gao X, Li X, Zhou P, Duan J. High-throughput construction of intron-containing hairpin RNA vectors for RNAi in plants. PLoS One. 2012;7(5):e38186.
4. Yuen ELH, Tumtas Y, King F, Ibrahim T, Chan LI, Evangelisti E, et al. A pathogen effector co-opts a host RabGAP protein to remodel pathogen interface and subvert defense-related secretion. Sci Adv. 2024;10(40):eado9516.
5. Foster SJ, Park TH, Pel M, Brigneti G, Sliwka J, Jagger L, et al. Rpi-vnt1.1, a Tm-2(2) homolog from Solanum venturii, confers resistance to potato late blight. Mol Plant Microbe Interact. 2009;22(5):589-600.
6. Gao C, Xu H, Huang J, Sun B, Zhang F, Savage Z, et al. Pathogen manipulation of chloroplast function triggers a light-dependent immune recognition. Proc Natl Acad Sci U S A. 2020;117(17):9613-20.
7. Sugihara Y, Kourelis J, Contreras MP, Pai H, Harant A, Selvaraj M, et al. Helper NLR immune protein NRC3 evolved to evade inhibition by a cyst nematode virulence effector. PLoS Genet. 2025;21(4):e1011653.
8. Bozkurt TO, Richardson A, Dagdas YF, Mongrand S, Kamoun S, Raffaele S. The Plant Membrane-Associated REMORIN1.3 Accumulates in Discrete Perihaustorial Domains and Enhances Susceptibility to Phytophthora infestans. Plant Physiol. 2014;165(3):1005-18.
