# Supplementary material for: A broad-host-range Rhizobium rhizogenes strain enables transient expression across diverse crop species
Source: PLoS Biol. 2026 Oct 15;24(10):e3004010. doi: 10.1371/journal.pbio.3004010 (PMC13671559; doi:10.1371/journal.pbio.3004010)
Supplement: Supplementary file 6 [file pbio.3004010.s012.docx]

| **Bacterial strain** | **Genotype** | **Selection used** |
| --- | --- | --- |
| *Agrobacterium fabrum* GV3101 | C58 Rif^R^ pMP90 (pTiC58ΔT Gen^R^) | 50 μg/mL rifampicin  50 μg/mL gentamycin |
| *A. fabrum* C58C1 | C58 Rif^R^ pGV2260 (pTiB6S3ΔT Carb^R^) pCH32^H^ | 50 μg/mL rifampicin  50 μg/mL carbenicillin |
| *A. fabrum* EHA105 | C58 Rif^R^ pEHA105 (pTiBO542ΔT) | 50 μg/mL rifampicin |
| *A. fabacearum* LBA4404 | Ach5 Rif^R^ pAL4404ΔT | 50 μg/mL rifampicin |
| *A. fabrum* AGL-1 | C58 Rif^R^ Carb^R^ pTiBO542ΔT | 50 μg/mL rifampicin  50 μg/mL carbenicillin |
| *Rhizobium rhizogenes* AS109 | A4 Rif^R^ ΔKan^R^ pRiA4ΔT | 50 μg/mL rifampicin |
