# Supplementary material for: A broad-host-range Rhizobium rhizogenes strain enables transient expression across diverse crop species
Source: PLoS Biol. 2026 Oct 15;24(10):e3004010. doi: 10.1371/journal.pbio.3004010 (PMC13671559; doi:10.1371/journal.pbio.3004010)
Supplement: Supplementary file 7 [file pbio.3004010.s016.docx]

REVIEWS:

Reviewer #1: The manuscript describes additional applications of a new Rhizobium rhizogenes strain AS109 for protein expression in diverse host plants, and cell biology and immunity experiments in previously recalcitrant fava bean (Vicia faba). AS109 was previously developed as an optimized strain for increasing transformation efficiency in Solanaceous plants. By thorough, quantitative, and statistically supported testing of RUBY expression in a broad number of species, this manuscript extends this utility to many new plant families and model systems. AS109 will be a critical new resource for experimental biology in diverse plant families. Biological novelty of the manuscript is limited because findings about NRC co-transfer, puncta formation, etc have already been shown in other model systems. A potentially novel haustorial interface revealed by AS109-expressed remorin is not explored in any detail. This is fine for a methods paper, and the potential for impact of AS109 for use in diverse experiments merits a broad audience publication.

We thank the reviewer for their positive comments regarding the utility of AS109-mediated expression, and for their suggested improvements to the manuscript.

My points 1-2 relate to making the strain's utility clear for other potential users, and I would not consider either one essential but instead recommended.

1. Mechanism mediating host range is not explored, but a small diversity screen could suggest NLR-based detection of AS109. Specifically, species showing lack of transformation are very likely due to the host plant immune response, which likely varies across different varieties of the same species. Evidence that this matters for AS109 host range could come from different varieties of a species showing qualitative difference in RUBY expression, consistent with the presence/abscence of an R gene.

If a quick variety screen of one of the non-expressing species (soybean, sesame, snapdragon, coffee, or quinoa) were to be performed and a positive variety were found, this would be very useful demonstration data that would encourage different research communities to screen their germplasm collections.

We agree with the reviewer that major differences in AS109 host range are likely due to immune responses, potentially corresponding to the NLR repertoire of the host species. In some cases, recalcitrance to AS109-mediated expression may also be due to the mechanical properties of the infiltrated leaves which can prevent the spread of the agroinfiltration.

We plan to perform further screening of recalcitrant species in the future; however, this is beyond the scope of our current manuscript given the large scale of these experiments and the requirement to obtain diverse varieties for screening.

2. All current claims about the ability to see specific localization are performed with heterologously expressed genes (StREM1.3 and NbNRC4). It would be impactful for future readers considering use of the strain to see localization of an endogenous legume gene. The Medicago community would be especially interested, and some classic genes like DMI2 or DMI3 could be tested for membrane / nuclear expression (or perhaps their Vicia faba homologs to stick with the fava-focused narrative). These experiments are almost certain to work, but the positive demonstration would still be impactful for convincing readers of the utility of transient expression.

Although it is feasible to transiently express endogenous genes, we believe that our manuscript already shows that AS109 facilitates cell biology study of diverse proteins (cytosolic GFP, transmembrane StREM1.3, and oligomeric NbNRC4). We can perform the suggested experiment, if necessary, but it is unlikely to be completed within the 1-month deadline for revisions.

Minor comments

- Fig 2: it would be useful to state the hairpin construct + vector in the figure and legend so that the reader does not have to hunt in S4 Table and references

For clarity, we have now added the construct details to the figure and legend of Fig 2.

- Pg 5 line 17: cite <https://www.cell.com/cell/abstract/S0092-8674(25)00578-1> for RTF

Thank you for highlighting this omission, we have now added the suggested reference (page 7, line 16).

- It is not clear how VfCC-NLR was selected. Basic phylogenetic analysis, closest soybean/other dicot homologs would be helpful.

We have now clarified our selection of *Vf*CC-NLR as a control for HR in text (page 6, lines 15-17):

“As a control for HR, we also expressed a CC-NLR cloned from faba bean (*Vf*CC-NLR) which has a canonical domain structure and is a member of the RPM1-like CC-NLR clade (S6 Fig) (Grant et al., 1995).”

We have also added an additional supplementary figure (S6 Fig) depicting the phylogenetic position of *Vf*CC-NLR within the annotated NLRs of the faba bean genome (see also Materials and Methods, Phylogenetic analysis).

Reviewer #2: The manuscript by King and colleagues describes testing of Rhizobium rhizogenes AS109 for leaf transient transformation across a number of plant species. It also demonstrates versatile use of the strain in faba bean, especially for the study of plant-pathogen interactions. Most of the results are clear and the strain will very likely prove of broad use in the plant science community. The manuscript appears to be written for a rather narrow plant-pathogen audience and some additional detail on figure interpretation and the genetic components used would be helpful.

We thank the reviewer for their suggestions to make our manuscript more accessible to the wider plant science community.

Specific suggestions

1. I suggest clarifying the origin and special properties of the strain by adding a few extra sentences in the introduction. Currently, Lopez-Agudelo et al. is required reading to acquire that information. The current manuscript is also quite similar to that paper, just focusing on other species. However, the broad usefulness balances out the potentially limited novelty.

We have now added an additional line in the introduction (page 2, lines 40-42) summarising the development of AS109 described in Lopez-Agudelo et al.

2. Strain availability. Please make it clear in the Data and materials availability statement how the strain can be accessed. Also, please make sure to make it easily accessible as this is critical for the value of the manuscript.

We have now clarified in the Data and materials availability statement that the AS109 strain can be requested from any one of the corresponding authors and will be provided upon completion of the required material transfer agreement (MTA) (page 13, lines 2-4). We are also in the process of depositing this strain in a public repository.

3. Fig. 3. Please clarify in the figure legend what DV, HA, 3E etc. indicate for the autoactive NLRs. What is panel E meant to illustrate? It looks like StREM1.3 is only introduced later in the text in relation to Fig. 5? Please clarify, also in the main text. Is there a substantial overlap between StREM1.3 and NbNRC4DV/L9E localisation in panel E? It does not look striking.

We thank the reviewer for suggesting these clarifications on the genetics and cell biology of NLRs. We have added an explanation of the NLR mutations in the legend of Fig 3. We have introduced the use of the *St*REM1.3 marker earlier in the main text and provided a more thorough explanation of the colocalisation (page 6, lines 21-28):

“To confirm the activation of heterologously expressed NRCs in faba bean, we examined the subcellular distribution of *Nb*NRC4 in its resting and active state. We co-expressed the WT and autoactive, non-cell-death-inducing NRC4 L9E mutant (*Nb*NRC4^DV/L9E^) with the *S. tuberosum* REMORIN marker (*St*REM1.3) in faba bean leaves. *St*REM1.3 is a plasma membrane marker which has been used to monitor helper NLR puncta at the cell surface in *N. benthamiana* (Ibrahim et al., 2026; Duggan et al., 2021; Bozkurt et al., 2014). Consistent with previous studies, the resting, inactive NRC4 showed a diffuse cytoplasmic distribution but, upon activation, formed membrane-associated puncta (Fig 3E).”

We have now added line micrographs to Fig 3E to demonstrate the quantitative colocalisation of RFP-*St*REM1.3 and *Nb*NRC4-GFP under resting and active conditions.

4. Fig. 5. Please provide a more detailed explanation of exactly what to look for here in panels B and C. There appears to be a lot of StRem1.3 signal, also in areas without hyphal contact? Also please briefly clarify why StRem1.3 is a relevant marker here, provide a reference and explain panel C including the line graphs in more detail in the figure legend.

We agree that greater explanation is needed in the section describing the novel *P. palmivora*-faba bean interface. We have added text clarifying the use of *St*REM1.3 as a marker of the extrahaustorial membrane and explaining our observations in more detail (page 8, lines 23-30):

In *N. benthamiana* cells, *St*REM1.3 accumulates at the plasma membrane and marks the specialised, host-derived membrane surrounding haustoria (feeding structures) of the oomycete pathogen *P. infestans* (Duggan et al., 2021; Bozkurt et al., 2014). Similarly, imaging faba bean cells at 1-2 days post-inoculation revealed clear accumulation of *St*REM1.3 at the plasma membrane and around *P. palmivora* haustoria, marking the extrahaustorial membrane (Fig 5B and 5C) (Bozkurt et al., 2014). We observed RFP-*St*REM1.3 both at the neck of *P. palmivora* haustoria and encasing the entire haustoria (Fig 5C).”

We have also added detail to the legend of Fig 5 to better explain the line graphs and our images of the *P. palmivora* extrahaustorial membrane.

Reviewer #3: In this manuscript, authors compare transient expression levels obtained after leaf infiltration with Rhizobium rhizogenes AS109 and several Agrobacterium strains classically used for this purpose. They show that AS109 was consistently superior (in terms of transient expression levels) to all other strains in most of wild and cultivated plant species tested. They also demonstrate that AS109 was suitable to perform several functional assays in Faba bean plants, showing that this system can be used for functional studies. Globally, it is an interesting study, and AS109 may prove to be a useful tool for plant biologists working with non-model plant species that are often considered as recalcitrant to T-DNA transfer from most Agrobacterium strains.

We thank the reviewer for their positive comments regarding the ability of AS109-mediated transient expression to enable functional assays in non-model plants.

Comments:

1: p.2, l. 22, to avoid confusion, it should be indicated that Rhizobium rhizogenes was previously known as Agrobacterium rhizogenes.

Thank you for highlighting this omission, we have corrected this in the main text (page 2, line 39).

2: p. 7, l. 30, why is R. rhizogenes grown on 523 medium as opposed to LB for Agrobacterium strains, is the medium used expected to affect the outcome in terms of transient expression?

For unknown reasons, *R. rhizogenes* AS109 is unable to grow on LB media. For the initial comparison of transient RUBY expression across species (Fig 1), all *Rhizobium* and *Agrobacterium* strains were grown on 523 media. For the secondary comparisons in faba bean and lettuce (Fig 2A-B; S5A-B Fig), the *Agrobacterium* strains were grown on LB media. Across all assays, *R. rhizogenes* AS109 mediated stronger RUBY expression than the *Agrobacterium* strains, and so we anticipate that growth medium has minimal effect on transient expression. We have now added these details to the Materials and Methods section to clarify this approach (page 10, lines 22-28).

3: In this study, authors measure the global transient expression levels (using RUBY) or, in Fig. 2C, GFP expression levels per cell. It would be interesting to have data showing percentage of expressing cells (or number of expressing cells per infiltrated surface area, maybe with a lower inoculum concentration). Usually, it is considered that the percentage of plant cells transformed depends on efficiency of T-DNA transfer while expression levels per cell may depend on other factors.

We thank the reviewer for this suggestion. Using the p35S::GFP microscopy data, we measured the proportion of cells in the field of view with GFP fluorescence for AS109 compared to GV3101 in faba bean (Fig 2E). Crucially, AS109-mediated expression leads to a significant increase in the proportion of cells showing fluorescence, suggesting that AS109 mediates both more efficient T-DNA transfer and stronger subsequent transgene expression.

4:It would be interesting to discuss whether the superiority of AS109 relies strictly on its Ri plasmid or if other features of the bacterial cells are involved (for example, would an Agrobacterium strain carrying pRiA4dT result in higher transformation efficiency?)

We thank the reviewer for this interesting point of discussion, which we have now included in the Discussion section (page 9, lines 12-24).

5: Table S4, the two first constructs have the same name (p35S::RUBY::t35S), are there any differences between them?

We thank the reviewer for catching this error in construct naming. Both RUBY constructs are under the 35S promoter, but the first contains the OCS terminator and the second the 35S terminator. The constructs were also cloned into different binary backbones (pICSL86922OD vs. pJK001c). We have now corrected these details in S4 Table.

Despite these minor construct differences, both RUBY expression assays show that AS109 mediates the strongest transient expression in faba bean and lettuce (Fig 1; Fig 2A-B; S5A-B Fig).
