# Supplementary material for: A broad-host-range Rhizobium rhizogenes strain enables transient expression across diverse crop species
Source: PLoS Biol. 2026 Oct 15;24(10):e3004010. doi: 10.1371/journal.pbio.3004010 (PMC13671559; doi:10.1371/journal.pbio.3004010)
Supplement: Peer review history

# Editor report: Decision Letter 0

**DOI:** 10.1371/journal.pbio.3004010.r001

**Authors:** Ankiit Ahluwalia

14 May 2026

Dear Dr Bozkurt,

Thank you for submitting your manuscript entitled "A broad-host-range Rhizobium rhizogenes strain enables transient expression across diverse crops and establishes functional assays in faba bean" for consideration as a Research Article by PLOS Biology.

Your manuscript has now been evaluated by the PLOS Biology editorial staff, as well as by an academic editor with relevant expertise, and I am writing to let you know that we would like to send your submission out for external peer review.

IMPORTANT: We think that your manuscript would be best considered as a Methods and Resources. No re-formatting is required at this stage, but please select "Methods and Resources" as the article type when you upload your additional metadata (see next paragraph).

However, before we can send your manuscript to reviewers, we need you to complete your submission by providing the metadata that is required for full assessment. To this end, please login to Editorial Manager where you will find the paper in the 'Submissions Needing Revisions' folder on your homepage. Please click 'Revise Submission' from the Action Links and complete all additional questions in the submission questionnaire.

Once your full submission is complete, your paper will undergo a series of checks in preparation for peer review. After your manuscript has passed the checks it will be sent out for review. To provide the metadata for your submission, please Login to Editorial Manager (https://www.editorialmanager.com/pbiology) within two working days, i.e. by May 18 2026 11:59PM.

If your manuscript has been previously peer-reviewed at another journal, PLOS Biology is willing to work with those reviews in order to avoid re-starting the process. Submission of the previous reviews is entirely optional and our ability to use them effectively will depend on the willingness of the previous journal to confirm the content of the reports and share the reviewer identities. Please note that we reserve the right to invite additional reviewers if we consider that additional/independent reviewers are needed, although we aim to avoid this as far as possible. In our experience, working with previous reviews does save time.

If you would like us to consider previous reviewer reports, please edit your cover letter to let us know and include the name of the journal where the work was previously considered and the manuscript ID it was given. In addition, please upload a response to the reviews as a 'Prior Peer Review' file type, which should include the reports in full and a point-by-point reply detailing how you have or plan to address the reviewers' concerns.

During the process of completing your manuscript submission, you will be invited to opt-in to posting your pre-review manuscript as a bioRxiv preprint. Visit http://journals.plos.org/plosbiology/s/preprints for full details. If you consent to posting your current manuscript as a preprint, please upload a single Preprint PDF.

Feel free to email us at plosbiology@plos.org if you have any queries relating to your submission.

Kind regards,

Ankiit

Ankiit Ahluwalia,

PLOS Biology

aahluwalia@plos.org

# Peer review: Decision Letter 1

**DOI:** 10.1371/journal.pbio.3004010.r002

**Authors:** Ankiit Ahluwalia

1 Jul 2026

Dear Dr Bozkurt,

Thank you for your patience while your manuscript "A broad-host-range Rhizobium rhizogenes strain enables transient expression across diverse crops and establishes functional assays in faba bean" went through peer-review at PLOS Biology. Your manuscript has now been evaluated by the PLOS Biology editors, an Academic Editor with relevant expertise, and by several independent reviewers.

In light of the reviews, which you will find at the end of this email, we are pleased to offer you the opportunity to address the [comments/remaining points] from the reviewers in a revision that we anticipate should not take you very long. We will then assess your revised manuscript and your response to the reviewers' comments with our Academic Editor aiming to avoid further rounds of peer-review, although we might need to consult with the reviewers, depending on the nature of the revisions.

In addition to these revisions, you may need to complete some formatting changes, which you will receive in a follow up email. A member of our team will be in touch with a set of requests shortly. If you do not receive a separate email within a few days, please assume that checks have been completed, and no additional changes are required.

We expect to receive your revised manuscript within 1 month. Please email us (plosbiology@plos.org) if you have any questions or concerns, or would like to request an extension.

At this stage, your manuscript remains formally under active consideration at our journal; please notify us by email if you do not intend to submit a revision so that we withdraw the manuscript.

**IMPORTANT - SUBMITTING YOUR REVISION**

Your revisions should address the specific points made by each reviewer. Please submit the following files along with your revised manuscript:

1. A 'Response to Reviewers' file - this should detail your responses to the editorial requests, present a point-by-point response to all of the reviewers' comments, and indicate the changes made to the manuscript.

*NOTE: In your point-by-point response to the reviewers, please provide the full context of each review. Do not selectively quote paragraphs or sentences to reply to. The entire set of reviewer comments should be present in full and each specific point should be responded to individually.

You should also cite any additional relevant literature that has been published since the original submission and mention any additional citations in your response.

2. In addition to a clean copy of the manuscript, please also upload a 'track-changes' version of your manuscript that specifies the edits made. This should be uploaded as a "Revised Article with Changes Highlighted " file type.

*Resubmission Checklist*

When you are ready to resubmit your revised manuscript, please refer to this resubmission checklist: https://plos.io/Biology_Checklist

To submit a revised version of your manuscript, please go to https://www.editorialmanager.com/pbiology/ and log in as an Author. Click the link labelled 'Submissions Needing Revision' where you will find your submission record.

Please make sure to read the following important policies and guidelines while preparing your revision:

*Published Peer Review*

Please note while forming your response, if your article is accepted, you may have the opportunity to make the peer review history publicly available. The record will include editor decision letters (with reviews) and your responses to reviewer comments. If eligible, we will contact you to opt in or out. Please see here for more details:

https://blogs.plos.org/plos/2019/05/plos-journals-now-open-for-published-peer-review/

*PLOS Data Policy*

Please note that as a condition of publication PLOS' data policy (http://journals.plos.org/plosbiology/s/data-availability) requires that you make available all data used to draw the conclusions arrived at in your manuscript. If you have not already done so, you must include any data used in your manuscript either in appropriate repositories, within the body of the manuscript, or as supporting information (N.B. this includes any numerical values that were used to generate graphs, histograms etc.). For an example see here: http://www.plosbiology.org/article/info%3Adoi%2F10.1371%2Fjournal.pbio.1001908#s5

*Blot and Gel Data Policy*

We require the original, uncropped and minimally adjusted images supporting all blot and gel results reported in an article's figures or Supporting Information files. We will require these files before a manuscript can be accepted so please prepare them now, if you have not already uploaded them. Please carefully read our guidelines for how to prepare and upload this data: https://journals.plos.org/plosbiology/s/figures#loc-blot-and-gel-reporting-requirements

*Protocols deposition*

To enhance the reproducibility of your results, we recommend that if applicable you deposit your laboratory protocols in protocols.io, where a protocol can be assigned its own identifier (DOI) such that it can be cited independently in the future. Additionally, PLOS ONE offers an option for publishing peer-reviewed Lab Protocol articles, which describe protocols hosted on protocols.io. Read more information on sharing protocols at https://plos.org/protocols?utm_medium=editorial-email&utm_source=authorletters&utm_campaign=protocols

Thank you again for your submission to our journal. We hope that our editorial process has been constructive thus far, and we welcome your feedback at any time. Please don't hesitate to contact us if you have any questions or comments.

Sincerely,

Ankiit

Ankiit Ahluwalia,

PLOS Biology

aahluwalia@plos.org

----------------------------------------------------------------

REVIEWS:

Reviewer #1: The manuscript describes additional applications of a new Rhizobium rhizogenes strain AS109 for protein expression in diverse host plants, and cell biology and immunity experiments in previously recalcitrant fava bean (Vicia faba). AS109 was previously developed as an optimized strain for increasing transformation efficiency in Solanaceous plants. By thorough, quantitative, and statistically supported testing of RUBY expression in a broad number of species, this manuscript extends this utility to many new plant families and model systems. AS109 will be a critical new resource for experimental biology in diverse plant families. Biological novelty of the manuscript is limited because findings about NRC co-transfer, puncta formation, etc have already been shown in other model systems. A potentially novel haustorial interface revealed by AS109-expressed remorin is not explored in any detail. This is fine for a methods paper, and the potential for impact of AS109 for use in diverse experiments merits a broad audience publication.

My points 1-2 relate to making the strain's utility clear for other potential users, and I would not consider either one essential but instead recommended.

1. Mechanism mediating host range is not explored, but a small diversity screen could suggest NLR-based detection of AS109. Specifically, species showing lack of transformation are very likely due to the host plant immune response, which likely varies across different varieties of the same species. Evidence that this matters for AS109 host range could come from different varieties of a species showing qualitative difference in RUBY expression, consistent with the presence/abscence of an R gene.

If a quick variety screen of one of the non-expressing species (soybean, sesame, snapdragon, coffee, or quinoa) were to be performed and a positive variety were found, this would be very useful demonstration data that would encourage different research communities to screen their germplasm collections.

2. All current claims about the ability to see specific localization are performed with heterologously expressed genes (StREM1.3 and NbNRC4). It would be impactful for future readers considering use of the strain to see localization of an endogenous legume gene. The Medicago community would be especially interested, and some classic genes like DMI2 or DMI3 could be tested for membrane / nuclear expression (or perhaps their Vicia faba homologs to stick with the fava-focused narrative). These experiments are almost certain to work, but the positive demonstration would still be impactful for convincing readers of the utility of transient expression.

Minor comments

- Fig 2: it would be useful to state the hairpin construct + vector in the figure and legend so that the reader does not have to hunt in S4 Table and references

- Pg 5 line 17: cite https://www.cell.com/cell/abstract/S0092-8674(25)00578-1 for RTF

- It is not clear how VfCC-NLR was selected. Basic phylogenetic analysis, closest soybean/other dicot homologs would be helpful.

Reviewer #2: The manuscript by King and colleagues describes testing of Rhizobium rhizogenes AS109 for leaf transient transformation across a number of plant species. It also demonstrates versatile use of the strain in faba bean, especially for the study of plant-pathogen interactions. Most of the results are clear and the strain will very likely prove of broad use in the plant science community. The manuscript appears to be written for a rather narrow plant-pathogen audience and some additional detail on figure interpretation and the genetic components used would be helpful.

Specific suggestions

1. I suggest clarifying the origin and special properties of the strain by adding a few extra sentences in the introduction. Currently, Lopez-Agudelo et al. is required reading to acquire that information. The current manuscript is also quite similar to that paper, just focusing on other species. However, the broad usefulness balances out the potentially limited novelty.

2. Strain availability. Please make it clear in the Data and materials availability statement how the strain can be accessed. Also, please make sure to make it easily accessible as this is critical for the value of the manuscript.

3. Fig. 3. Please clarify in the figure legend what DV, HA, 3E etc. indicate for the autoactive NLRs. What is panel E meant to illustrate? It looks like StREM1.3 is only introduced later in the text in relation to Fig. 5? Please clarify, also in the main text. Is there a substantial overlap between StREM1.3 and NbNRC4DV/L9E localisation in panel E? It does not look striking.

4. Fig. 5. Please provide a more detailed explanation of exactly what to look for here in panels B and C. There appears to be a lot of StRem1.3 signal, also in areas without hyphal contact? Also please briefly clarify why StRem1.3 is a relevant marker here, provide a reference and explain panel C including the line graphs in more detail in the figure legend.

Reviewer #3: In this manuscript, authors compare transient expression levels obtained after leaf infiltration with Rhizobium rhizogenes AS109 and several Agrobacterium strains classically used for this purpose. They show that AS109 was consistently superior (in terms of transient expression levels) to all other strains in most of wild and cultivated plant species tested. They also demonstrate that AS109 was suitable to perform several functional assays in Faba bean plants, showing that this system can be used for functional studies. Globally, it is an interesting study, and AS109 may prove to be a useful tool for plant biologists working with non-model plant species that are often considered as recalcitrant to T-DNA transfer from most Agrobacterium strains.

Comments:

1: p.2, l. 22, to avoid confusion, it should be indicated that Rhizobium rhizogenes was previously known as Agrobacterium rhizogenes.

2: p. 7, l. 30, why is R. rhizogenes grown on 523 medium as opposed to LB for Agrobacterium strains, is the medium used expected to affect the outcome in terms of transient expression?

3: In this study, authors measure the global transient expression levels (using RUBY) or, in Fig. 2C, GFP expression levels per cell. It would be interesting to have data showing percentage of expressing cells (or number of expressing cells per infiltrated surface area, maybe with a lower inoculum concentration). Usually, it is considered that the percentage of plant cells transformed depends on efficiency of T-DNA transfer while expression levels per cell may depend on other factors.

4:It would be interesting to discuss whether the superiority of AS109 relies strictly on its Ri plasmid or if other features of the bacterial cells are involved (for example, would an Agrobacterium strain carrying pRiA4dT result in higher transformation efficiency?)

5: Table S4, the two first constructs have the same name (p35S::RUBY::t35S), are there any differences between them?

# Author comment: Author response to Decision Letter 2

**DOI:** 10.1371/journal.pbio.3004010.r003

17 Jul 2026

Attachment Submitted filename: AS109_Response_to_reviewers.docx

# Peer review: Decision Letter 2

**DOI:** 10.1371/journal.pbio.3004010.r004

**Authors:** Ankiit Ahluwalia

8 Sep 2026

Dear Dr Bozkurt,

Thank you for the submission of your revised Methods and Resources "A broad-host-range Rhizobium rhizogenes strain enables transient expression across diverse crop species" for publication in PLOS Biology. On behalf of my colleagues and the Academic Editor, Xinnian Dong, I am pleased to say that we can in principle accept your manuscript for publication, provided you address any remaining formatting and reporting issues. These will be detailed in an email you should receive within 2-3 business days from our colleagues in the journal operations team; no action is required from you until then. Please note that we will not be able to formally accept your manuscript and schedule it for publication until you have completed any requested changes.

IMPORTANT: After discussion among the team we think that your Title will be more appealing and accessible without the last phrase. We have therefore shortened your Title to "A broad-host-range Rhizobium rhizogenes strain enables transient expression across diverse crop species." We also note that the additional functional proof-of-principle in fava bean is clearly mentioned in the Abstract for more interested readers. I hope that you understand our reasons for doing this.

Please take a minute to log into Editorial Manager at http://www.editorialmanager.com/pbiology/, click the "Update My Information" link at the top of the page, and update your user information to ensure an efficient production process.

PRESS

We frequently collaborate with press offices. If your institution or institutions have a press office, please notify them about your upcoming paper at this point, to enable them to help maximise its impact. If the press office is planning to promote your findings, we would be grateful if they could coordinate with biologypress@plos.org. If you have previously opted in to the early version process, we ask that you notify us immediately of any press plans so that we may opt out on your behalf.

We also ask that you take this opportunity to read our Embargo Policy regarding the discussion, promotion and media coverage of work that is yet to be published by PLOS. As your manuscript is not yet published, it is bound by the conditions of our Embargo Policy. Please be aware that this policy is in place both to ensure that any press coverage of your article is fully substantiated and to provide a direct link between such coverage and the published work. For full details of our Embargo Policy, please visit http://www.plos.org/about/media-inquiries/embargo-policy/.

Thank you again for choosing PLOS Biology for publication and supporting Open Access publishing. We look forward to publishing your study.

Sincerely,

Ankiit Ahluwalia,

PLOS Biology

aahluwalia@plos.org
